# Experimental and real‐world evidence supporting the repurposing of drugs targeting proteotoxicity and inflammation to treat Alzheimer's disease

**DOI:** 10.1002/alz70860_103570

**Published:** 2025-12-23

**Authors:** Rachel R Litke, Arushi Arora, Yihan Wang, Melissa D Aldridge, Fred C Ko, Sven Sandin, Mary Sano, Abraham Reichenberg, Charles V Mobbs

**Affiliations:** ^1^ Icahn School of Medicine at Mount Sinai, New York, NY, USA; ^2^ Icahn School Of Medicine at Mount Sinai, New York, NY, USA; ^3^ Karolinska Institutet, Stockholm, Sweden; ^4^ James J. Peters VA Medical Center, Bronx, NY, USA

## Abstract

**Background:**

Alzheimer's Disease and Related Dementia (ADRD) affects 10.9% of people above 65 years old in the US. Disease modifying and symptomatic therapies for ADRD have marginal clinical value; thus finding effective treatments is paramount. Inflammation and proteotoxicity are two hallmarks of aging implicated in the pathophysiology of ADRD. We previously screened 2700 FDA approved drugs in two models of ADRD (C. elegans and microglia) for their ability to reduce inflammation and proteotoxicity and identified phenothiazines among the most protective class of drugs. This study aimed to use a pharmacoepidemiological approach with Medicare claims data to assess if phenothiazines could be repurposed in humans to treat ADRD.

**Methods:**

To test our hypothesis that phenothiazines were protective for ADRD, we examined 1 year of cross‐sectional Medicare fee‐for‐service claims data of 1 million Medicare beneficiaries >65 years. We performed multivariable analysis adjusted for demographics and ADRD risk factors to evaluate the association between ADRD prevalence and phenothiazine exposure. Our principal outcome was ADRD as defined by a modified Bynum algorithm (unavailable Amyloid documentation in our dataset).

**Results:**

33,600 (3.5%) beneficiaries (70.0% women) were exposed to the drugs of interest. The mean age of exposed beneficiaries was 74 years (SD 6.6) versus 75 years (SD 7.3) for unexposed beneficiaries. The multivariable analysis revealed a significantly lower ADRD prevalence in phenothiazines exposed (≥1phenothiazine) vs unexposed beneficiaries (Incident Rate Ratio 0.9; 95%CI 0.9‐0.97). Some protective phenothiazines in our dataset included amitriptyline (IRR 0.8; 95%CI 0.8‐0.9) and prochlorperazine (IRR 0.8; 95%CI 0.7‐0.8).

**Conclusions:**

By leveraging real‐world data with Medicare fee‐for‐service claims, phenothiazine exposure was associated with a lower risk of ADRD in US individuals >65 years, suggesting a potential benefit of phenothiazines in reducing the risk of ADRD. This study is strengthened by real world data including selection of a target population and studying the interaction of age and ADRD in a short timespan. A limitation of our study is the lack of information on amyloid documentation inherent with the nature of administrative claims data from CMS. Further studies are warranted to determine whether the observed association between phenothiazine exposure and protection against ADRD in humans is confirmed longitudinally

